# Saposhnikoviae Radix Enhanced the Angiogenic and Anti-Inflammatory Effects of Huangqi Chifeng Tang in a Rat Model of Cerebral Infarction

**DOI:** 10.1155/2021/4232708

**Published:** 2021-09-21

**Authors:** Qiu-Yue Wang, Na Zhang, Shu-Yu Liu, Xi-Hong Jiang, Shu-Min Liu

**Affiliations:** ^1^Institute of Traditional Chinese Medicine, Heilongjiang University of Chinese Medicine, Harbin 150040, China; ^2^Drug Safety Evaluation Centre, Heilongjiang University of Chinese Medicine, Harbin 150040, China

## Abstract

Huangqi Chifeng Tang (HQCFT), a traditional Chinese formula of three herbs, has been used to treat cerebral infarction (CI). Saposhnikoviae Radix (SR) was designed as a guiding drug for HQCFT to improve its angiogenic and anti-inflammatory effects. In this study, TTC staining was used to detect the area of CI. H&E staining was used to detect the histopathologic changes in the cerebral tissue. Western blotting was performed to detect the protein expression of NLRP3, caspase 1, IL-1*β*, IL-6, TNF-*α*, MMP-9, VEGF, and VEGFR2 in cerebral tissue. Immunohistochemistry was used to detect the protein expression of MMP-9, VEGF, and VEGFR2. The contents of HIF-1*α*, NLRP3, caspase 1, IL-1*β*, IL-6, and TNF-*α* in the serum were determined by ELISA. Our study showed that HQCFT and HQCFT-SR could improve the pathological condition and reduce the infarcted area of the brain tissue in a rat model. In addition, HQCFT and HQCFT-SR significantly decreased the expression levels and serum contents of NLRP3, caspase 1, IL-1*β*, IL-6, and TNF-*α*; increased the expression levels of the VEGF and VEGFR2 proteins; and obviously reduced the serum content of HIF-1*α*. Importantly, the cytokines in brain tissue and serum from the HQCFT group exhibited better efficacy than those from the HQCFT-SR group. HQCFT exerted significant angiogenic and anti-inflammatory effects in rats subjected to middle cerebral artery occlusion (MCAO); these effects can be attributed to the guiding and enhancing effect of SR.

## 1. Introduction

Huangqi Chifeng Tang (HQCFT), a traditional Chinese herbal formula, was documented in “Yi Lin Gai Cuo” (Correcting the Errors in Medical Works) by Wang Qingren in 1830 AD [1]. It is composed of three Chinese medicines: Astragali Radix (AR; Chinese name: Huangqi), Paeoniae Radix (PR; Chinese name: Chishao), and Saposhnikoviae Radix (SR; Chinese name: Fangfeng). AR has anti-inflammatory, neuroprotective, angiogenic, and immunomodulatory effects [2–4], which play an important role in HQCFT. PR has an auxiliary effect on AR, and SR is used as an adjuvant and messenger drug to improve the overall therapeutic effects of AR and PR. Modern pharmacological research has shown that modified HQCFT can improve proteinuria and protect against glomerulus and podocyte injury by repressing excessive autophagy through the PI3K/mTOR signaling pathway [5].

SR, derived from the root of *Saposhnikovia divaricata* (Turcz.) Schischk., is commonly used to treat colds, headaches, rheumatic arthralgia, and rubella-associated itching. It has been reported that SR can increase the distribution of atractylenolide I, paeoniflorin, and hesperidin in the liver, spleen, brain, and small intestine; reduce the distribution of these components in the blood and lungs; and improve drug efficacy by promoting entry into the brain [6]. The absorption and pharmacokinetic behavior of the three main components (cimicifugoside, 4-O-beta-d-glucosyl-5-O-methylvisamminol, and sec-O-glucosylhamaudol) of SR are superior to those of the other major active components and may play a guiding role in the overall therapeutic effects of Yupingfeng (YPF) [7]. Moreover, the guiding effect of SR has been used in a variety of ancient prescriptions, such as HQCFT, Baizhu Saoyao San, Tongxie Yaofang, and YPF [6, 8].

SR often serves as a guiding drug, which improves the therapeutic effects of other drugs or components, especially in the respiratory tract and brain diseases. Nevertheless, little is known about the guiding effect of SR. In the present study, we investigated the pharmacodynamic effect of the SR in HQCFT in the treatment of cerebral infarction (CI) by investigating its angiogenic and anti-inflammatory effects.

## 2. Materials and Methods

### 2.1. Chemicals and Reagents

2,3,5-Triphenyltetrazolium chloride (TTC) solution was purchased from Beijing Solarbio Science & Technology Co., Ltd. (Beijing, China). ELISA kits for hypoxia-inducible factor 1*α* (HIF-1*α*), NLR family pyrin domain-containing 3 (NLRP3), caspase 1, interleukin-1*β* (IL-1*β*), interleukin-6 (IL-6), tumor necrosis factor-*α* (TNF-*α*), and matrix metalloproteinase-9 (MMP-9) were purchased from Nanjing Jiancheng Bioengineering Institute (Nanjing, China).

### 2.2. Plant Materials and Extraction

Astragali Radix (the bark of *Astragalus membranaceus* (Fisch.) Bge., No. 20190401), Radix Paeoniae Rubra (the bark of *Paeonia lactiflora* Pall., No. 20191001), and Saposhnikoviae Radix (*Saposhnikovia divaricata* (Turcz.) Schischk., No. 20190402) were all obtained from Heilongjiang Xiushengtang Medicinal Materials Co., Ltd. (Heilongjiang, China) and authenticated by Prof. Wang Zhen Yue (Hei long Jiang, University of Chinese Medicine). The voucher specimens (hlj-201,508) were stored in the Herbarium of Heilongjiang University of Chinese Medicine (Heilongjiang, China). All herbs in HQCFT were soaked in distilled water (1 : 10, *w*/*v*) for 1 h before extraction and then extracted three times for 1.5 h each. The extraction solutions were collected and concentrated to a suitable volume by decompression, and the residue was deposited in a vacuum freeze dryer for 48 hours. In addition, the extraction method for HQCFT without SR (HQCFT-SR) was the same as that used for HQCFT. The extraction ratios of HQCFT and HQCFT-SR were 38.53% and 37.31% (w/w), respectively.

### 2.3. Animals

Forty-eight adult male SD rats (240 ± 20 g) were supplied by Shenyang Changsheng Biotechnology Co., Ltd. (China). The rats were housed six per cage in the animal house of the Institute of Chinese Medicine, Heilongjiang University of Chinese Medicine, with a controlled room temperature (24 ± 2°C) and relative humidity (55 ± 5%). The rats were allowed to acclimate for 7 days prior to the experiments, with a 12 h light and dark cycle (lights on from 08:00 a.m. to 08:00 p.m.) and given food and water ad libitum. The animal studies received ethical approval in accordance with the Legislation on the Protection of Animals Used for Experimental Purposes (Directive 86/609/EEC), and the experiments were approved by the Institutional Animal Care Committee (Permission No. 20,190,921).

### 2.4. Preparation of the Middle Cerebral Artery Occlusion (MCAO) Model

The MCAO rat model was established according to previous studies [9, 10]. Briefly, rats were deeply anesthetized with 3% pentobarbital sodium (45 mg/kg) by intraperitoneal injection. A midline incision was made for blunt separation of the right common carotid artery (CCA), external carotid artery (ECA), and internal carotid artery (ICA). A 40 mm nylon thread plug (RWD Life Science Co., Ltd., Shenzhen, China) was inserted approximately 18 ± 2 mm into the ICA from the CCA. The proximal and distal ends of the CCA were ligated to prevent bleeding, and the wound was sutured. The rats were placed at a constant temperature of 37°C until they regained consciousness.

### 2.5. Drug Administration

Forty-eight SD rats were randomly divided into 4 groups: (1) the model group: rats subjected to MCAO were administered normal saline; (2) the sham operation group: rats subjected to MCAO were administered normal saline; (3) the HQCFT group (1.6 g/kg): rats subjected to MCAO were administered HQCFT, that is, AR : RPR : SR = 10 : 3 : 2; and (4) the HQCFT-SR group (1.31 g/kg): rats subjected to MCAO were administered HQCFT without SR, that is, AR : RPR : SR = 10 : 3 : 0. All treatments were administered every 24 h for 14 days.

### 2.6. Blood and Brain Tissue Sample Collection

After 14 days of treatment, all rats were fasted with free access to water for 12 h. Then, the rats were deeply anesthetized with 3% pentobarbital sodium (45 mg/kg) by intraperitoneal injection. Whole blood samples were collected from the abdominal artery prior to rat sacrifice. Fresh blood was allowed to stand for approximately 30 min at ambient temperature (22–25°C), and serum was obtained after centrifugation at 3500 g for 15 minutes at 4°C. Simultaneously, one portion of the whole brain tissue was rapidly separated and frozen at −20°C for 30 min, and the other portions were stored in 4% paraformaldehyde and liquid nitrogen.

### 2.7. Quantification of Cerebral Infarct Volume

After blood collection, the auricula dextra of the rats was dissected, and prechilled normal saline was perfused from the left ventricle. Then, the fresh brain tissue was eviscerated and frozen at −20°C for 30 min. The brain was carefully cut into 2.0 mm thick serial cross sections from front to back and quickly incubated in 2% TTC solution at 37°C for 30 min, protected from exposure to light. Finally, the brain slices were fixed in 4% paraformaldehyde at room temperature for 12 h. The rat CI area was measured with Image-Pro Plus software, and the equation used for calculations was as follows:

(1) $$\begin{array}{c} \text{Brain infarction volume }\left(\text{\%}\right)=\frac{\left(\text{Ai}1+\cdots +\text{Ai}n\right)T}{\left(\text{A}t1+\cdots +\text{At}n\right)T}\times 100\%. \end{array}$$

Ai represents the CI area, At represents the whole cerebral area, and *T* represents the thickness of each slice.

### 2.8. Hematoxylin and Eosin (H&E) Staining

H&E staining was performed to observe the histopathological changes in rat cerebral tissues. Briefly, rat brain tissue was fixed in 4% paraformaldehyde for 48 h and prepared into 5 *μ*m paraffin slices. Then, the sections were dewaxed with xylene and dehydrated in an alcohol gradient. Hematoxylin and eosin were used to stain the sections, and pathological features of the brain tissue were examined under a 400× optical microscope (Olympus, Japan).

### 2.9. Western Blot Analysis

Briefly, the brain tissues from the left hemisphere were washed twice with PBS and disrupted with RIPA lysis buffer. Then, the tissue suspension was centrifuged at 12,000 rpm for 15 min at 4°C, and the supernatant was used to determine the concentration of the sample with a BCA protein assay kit (Beyotime, China). Equal amounts of samples were separated by SDS-PAGE and electrotransfered to polyvinylidene difluoride membranes. Subsequently, the membranes were blocked with 5% skim milk for 1 h at room temperature and incubated overnight at 4°C with the indicated primary antibodies against NLRP3 (1 : 500, Bios), caspase 1 (1 : 1000, Abcam), IL-1*β* (1 : 500, Abcam), IL-6 (1 : 1000, Abcam), TNF-*α* (1 : 500, Gene Tex), MMP-9 (1 : 500, Bios), vascular endothelial growth factor (VEGF) (1 : 500, Bios), vascular endothelial growth factor receptor 2 (VEGFR2) (1 : 1000, CST), and glyceraldehyde-3-phosphate dehydrogenase (GAPDH) (1 : 2500, Abcam). After rinsing with TBST three times, the membranes were incubated with the appropriate horseradish peroxidase-conjugated secondary antibodies (1 : 1000, Abcam) for 1 h at 37°C. Chemiluminescence (ECL) kits were used to visualize the membranes, and a Syngene Tanon 5200 imaging system (Tanon, China) was used for imaging. The optical density of the bands was quantified using ImageJ software and corrected according to the corresponding GAPDH level.

### 2.10. Immunohistochemical Analysis

The brain tissue was fixed in 4% paraformaldehyde for 48 h and prepared into 5 *μ*m paraffin sections for immunohistochemical analysis. In brief, the endogenous peroxidase activity of the slices was quenched by incubation with 3% H_2_O_2_ for 10 min and blocking with goat serum for 30 min. Subsequently, MMP-9, VEGF, and VEGFR2 antibodies were added to the slices at 4°C overnight. The slices were incubated at room temperature for 40 min, rinsed with PBS three times, incubated with the secondary antibodies at 37°C for 60 min, and visualized with 3,3-diaminobenzidine. The images were observed under high magnification (400×), and the optical density of positive cells was analyzed with ImageJ software.

### 2.11. Enzyme-Linked Immunosorbent Assay (ELISA)

The serum contents of HIF-1*α*, NLRP3, caspase 1, IL-1*β*, IL-6, and TNF-*α* were detected using ELISA kits following the manufacturer's protocol. The sample absorbance was assessed at 450 nm using a Multifunctional Microplate Scanner (TECAN, Infinite M200 PRO, Switzerland). The contents of HIF-1*α*, NLRP3, caspase 1, IL-1*β*, IL-6, and TNF-*α* were calculated by using a standard curve.

### 2.12. Statistical Analysis

Experimental data are expressed as the mean ± standard deviation (SD). Statistical significance was determined using Student's *t*-tests and one-way analysis of variance (ANOVA) followed by Fisher's LSD multiple comparisons test. A value of *p* < 0.05 was considered to indicate statistical significance.

## 3. Results

### 3.1. Effect of SR on the Infarction Area in the Brain Tissue of Rats Subjected to MCAO

**Figure 1:**
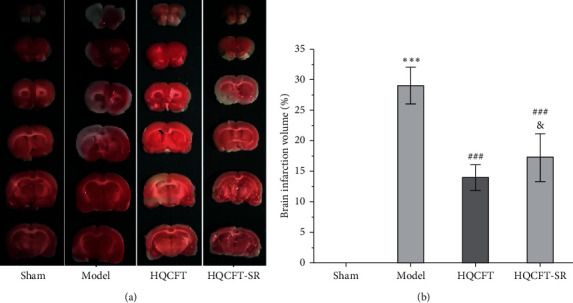
TTC staining to determine the infarction volume in rat brain tissue. (a) Images of TTC staining of rat brain tissue. The infarction area is white, and the normal area is red. (b) Analysis of the cerebral infarction volume results in rats. The pictures shown are representative of six independent samples. Data are expressed as the mean ± SD (*n* = 6). ^∗∗∗^*p* < 0.001 vs. the sham operation group, ^###^*p* < 0.001 vs. the model group, and ^&^*p* < 0.05 vs. the HQCFT group.

TTC can be reduced to 1,3,5-triphenylformazan by dehydrogenase in normal brain tissue and is red, while infarcted cells lose vitality and are white or ivory [11]. Figures 1(a) and 1(b) show that no infarction area was observed in the sham group (0.000 ± 0.000). In contrast, the infarction ratio in the model group (29.065 ± 3.042) was markedly increased compared with that in the sham group. The HQCFT (13.993 ± 2.126) and HQCFT-SR (17.285 ± 3.908) treatment groups had significantly decreased infarction ratios compared with that of the model group. The infarction ratio in the HQCFT group was also obviously lower than that in the HQCFT-SR group (*p* < 0.05), indicating that the SR in HQCFT could attenuate infarction in the brain tissue of rats subjected to MCAO.

### 3.2. Effect of SR on Histopathological Changes in the Brain Tissue of Rats Subjected to MCAO

**Figure 2:**
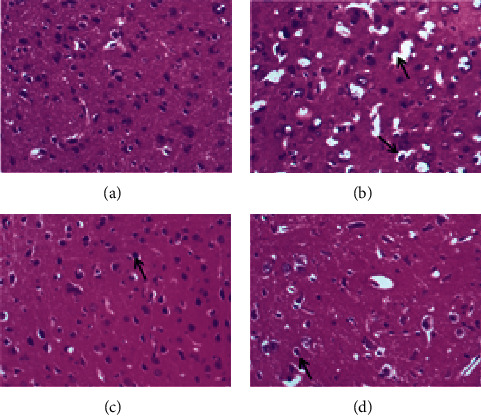
Histopathological analysis of rat brain tissue. (a) The sham group and (b) model group were administered normal saline for 14 days. (c) The HQCFT group and (d) HQCFT-SR group were treated with HQCFT extract and HQCFT without SR for 14 days, respectively, and the dose was 1.6 g/kg and 1.31 g/kg daily, respectively. The slices were magnified 200 times, and the pictures shown are representative of three independent samples. The arrows in Figure 2(b) indicate edema and vacuolization; the arrows in Figures 2(c) and 2(d) indicate neuronal cells.

The histopathological changes of rats were assessed after 14 days of treatment, and H&E staining of slices was performed to evaluate the severity of CI. As shown in Figure 2, in the sham group, no obvious edema, vacuolization, or neuronal injury was observed in the brain tissue. In contrast, the neurons in rats subjected to MCAO were damaged and necrotic, with severe edema and vacuolization. Local gliocytes aggregated, and the cell morphology changed. The brain tissue of the HQCFT and HQCFT-SR treatment groups showed an improved pathological condition, accompanied by less edema, vacuolization, and nuclear pyknosis. In particular, the neuronal cells in the HQCFT group were clearer and more complete than those in the HQCFT-SR group.

### 3.3. Effect of SR on NLRP3, Caspase 1, and IL-1*β* Protein Expression in the Brains of Rats Subjected to MCAO

**Figure 3:**
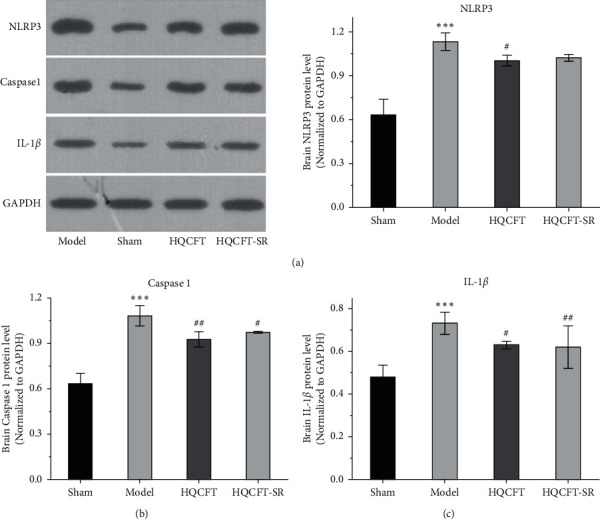
Effects of SR on the protein expression levels of NLRP3, caspase 1, and IL-1*β* in the brain tissue of rats subjected to MCAO. (a) NLRP3 expression in rat brain tissue, (b) caspase 1 expression in rat brain tissue, and (c) IL-1*β* expression in rat brain tissue. The sham group and model group were administered normal saline for 14 days. The HQCFT group and HQCFT-SR group were treated with HQCFT extract and HQCFT without SR for 14 days, respectively, and the dose was 1.6 g/kg and 1.31 g/kg daily, respectively. Data are expressed as the mean ± SD (*n* = 3). ^∗∗∗^*p* < 0.001 vs. the sham operation group, ^#^*p* < 0.05 and ^##^*p* < 0.01 vs. the model group.

As NLRP3, caspase 1, and IL-1*β* have been reported to be involved in inflammatory responses [12, 13], this study examined the protein expression levels of NLRP3, caspase 1, and IL-1*β* in rat brain tissue by Western blot analysis. As shown in Figure 3(a), the caspase 1 protein expression level in the model group (1.083 ± 0.067) was significantly higher than that in the sham group (0.636 ± 0.064). HQCFT (0.930 ± 0.050) and HQCFT-SR (0.977 ± 0.006) treatment significantly decreased the expression level compared with that in the model group. However, there was no significant difference between the HQCFT and HQCFT-SR groups (*p* > 0.05).

As shown in Figure 3(b), the expression level of NLRP3 in the model group (1.133 ± 0.060) was increased compared with that in the sham group (0.633 ± 0.105). After treatment with HQCFT (1.003 ± 0.038), NLRP3 protein expression was obviously decreased. However, the level of NLRP3 in the HQCFT-SR group (1.023 ± 0.023) was not significantly different from that in the model group (*p* > 0.05), and no significant differences were noted between the HQCFT and HQCFT-SR groups (*p* > 0.05).

As shown in Figure 3(c), in the model group (1.137 ± 0.042), the protein content of IL-6 was higher than that in the sham group (0.730 ± 0.079). However, HQCFT (0.980 ± 0.056) and HQCFT-SR (0.983 ± 0.042) treatment significantly decreased the expression level of IL-6 compared with that in the model group. No significant difference was observed between the HQCFT and HQCFT-SR groups (*p* > 0.05).

### 3.4. Effect of SR on TNF-*α* and IL-6 Protein Expression in the Brains of Rats Subjected to MCAO

**Figure 4:**
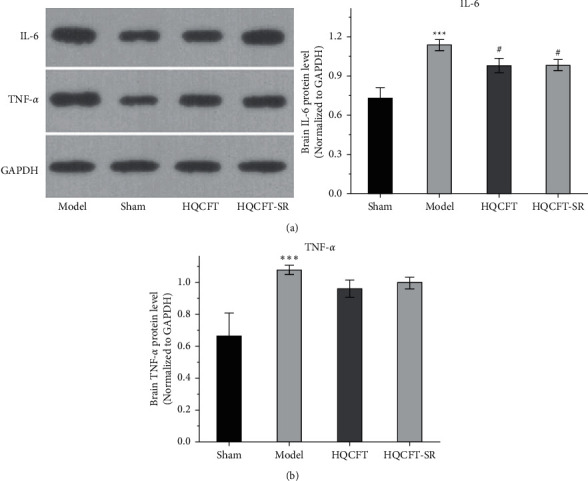
Effects of HQCFT and HQCFT-SR on the protein expression of IL-6 and TNF-*α* in the brain tissue of rats subjected to MCAO. (a) IL-6 expression in rat brain tissue and (b) TNF-*α* expression in rat brain tissue. The sham group and model group were administered normal saline for 14 days. The HQCFT group and HQCFT-SR group were treated with HQCFT extract and HQCFT without SR for 14 days, respectively, and the dose was 1.6 g/kg and 1.31 g/kg daily, respectively. Data are expressed as the mean ± SD (*n* = 3). ^∗∗∗^*p* < 0.001 vs. the sham operation group, ^##^*p* < 0.01 and ^###^*p* < 0.001 vs. the model group.

TNF-*α* and IL-6 are important inflammatory factors in the pathogenesis of CI. As shown in Figure 4(a), the expression level of IL-6 in the model group (0.733 ± 0.051) was higher than that in the sham group (0.480 ± 0.056), while IL-6 expression was significantly downregulated in both the HQCFT (0.630 ± 0.017) and HQCFT-SR (0.620 ± 0.017) treatment groups, with no significant difference between these two groups (*p* > 0.05).

As shown in Figure 4(b), the TNF-*α* protein contents were 0.663 ± 0.144, 1.077 ± 0.029, 0.960 ± 0.053, and 0.997 ± 0.035 in the sham, model, HQCFT, and HQCFT-SR groups, respectively. Compared with that in the sham group, the TNF-*α* protein content increased by 62.44% (*p* < 0.001) in the model group. However, there was no statistically significant difference compared with the model group, and no significant difference was observed between the HQCFT and HQCFT-SR groups (*p* > 0.05).

### 3.5. Effect of SR on MMP-9, VEGF, and VEGFR2 Protein Expression in the Brains of Rats Subjected to MCAO

**Figure 5:**
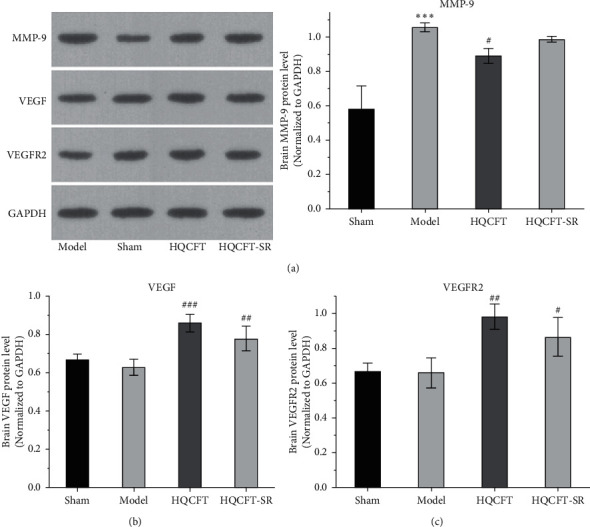
Effects of HQCFT and HQCFT-SR on the protein expression levels of MMP-9, VEGF, and VEGFR2 in the brain tissue of rats subjected to MCAO. (a) MMP-9 expression in rat brain tissue, (b) VEGF expression in rat brain tissue, and (c) VEGFR2 expression in rat brain tissue. The sham group and model group were administered normal saline for 14 days. The HQCFT group and HQCFT-SR group were treated with HQCFT extract and HQCFT without SR for 14 days, respectively, and the dose was 1.6 g/kg and 1.31 g/kg daily, respectively. Data are expressed as the mean ± SD (*n* = 3). ^∗∗∗^*p* < 0.001 vs. the sham operation group, ^#^*p* < 0.05, ^##^*p* < 0.01, and ^###^*p* < 0.001 vs. the model group.

The expression of MMP-9, VEGF, and VEGFR2 plays an important role in neuroinflammation and angiogenesis [14, 15]. As shown in Figure 5(a), compared with that in the sham group (0.580 ± 0.135), the protein expression level of MMP-9 in the model group (1.057 ± 0.025) was increased significantly. Compared with the model group, the HQCFT (0.890 ± 0.044) and HQCFT-SR (0.987 ± 0.015) treatment groups showed marked downregulation of MMP-9 expression levels in brain tissue, but there was no significant difference between the two treatment groups (*p* > 0.05).

As shown in Figure 5(b), the VEGF expression levels were 0.670 ± 0.026, 0.627 ± 0.042, 0.857 ± 0.045, and 0.777 ± 0.065 in the sham, model, HQCFT, and HQCFT-SR groups, respectively. Compared with that in the sham group, VEGF protein expression was decreased by 6.42% (*p* > 0.05) in the model group. Compared with that in the model group, VEGF protein expression was increased by 33.68% (*p* < 0.001) and 23.92% (*p* < 0.01) in the HQCFT and HQCFT-SR groups, respectively. The level of VEGF in the HQCFT-SR group was not significantly different from that in the HQCFT group (*p* > 0.05).

As shown in Figure 5(c), the VEGFR2 levels were 0.667 ± 0.049, 0.660 ± 0.087, 0.983 ± 0.072, and 0.867 ± 0.110 in the sham, model, HQCFT, and HQCFT-SR groups, respectively. Compared with that in the sham group, VEGFR2 protein expression was decreased by 1.05% (*p* > 0.05) in the model group. Compared with that in the model group, VEGFR2 protein expression was increased by 48.94% (*p* < 0.01) and 31.36% (*p* < 0.05) in the HQCFT and HQCFT-SR groups, respectively, and there was no significant difference between these two groups (*p* > 0.05).

### 3.6. Effect of SR on MMP-9, VEGF, and VEGFR2 Protein Expression Determined by Immunohistochemical Analysis

**Figure 6:**
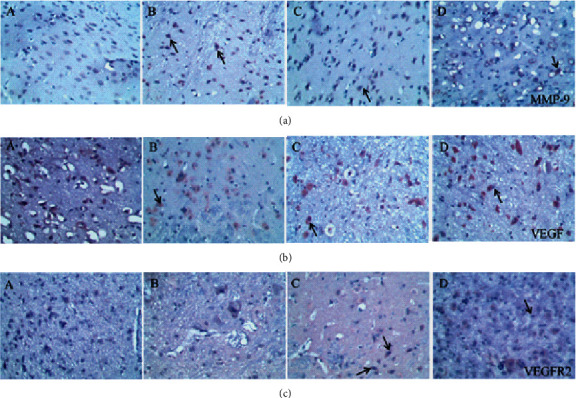
Immunohistochemical staining of (a) MMP-9, (b) VEGF, and (c) VEGFR2 in rat brain tissue. (A) The sham group and (B) model group were administered normal saline for 14 days. (C) The HQCFT group and (D) HQCFT-SR group were treated with HQCFT extract and HQCFT without SR for 14 days, respectively, and the dose was 1.6 g/kg and 1.31 g/kg daily, respectively. The sections were magnified 200 times, and gray MMP-9-, VEGF-, and VEGFR2-positive cells were found in the rat brain tissue. The pictures shown are representative of three independent samples.

**Figure 7:**
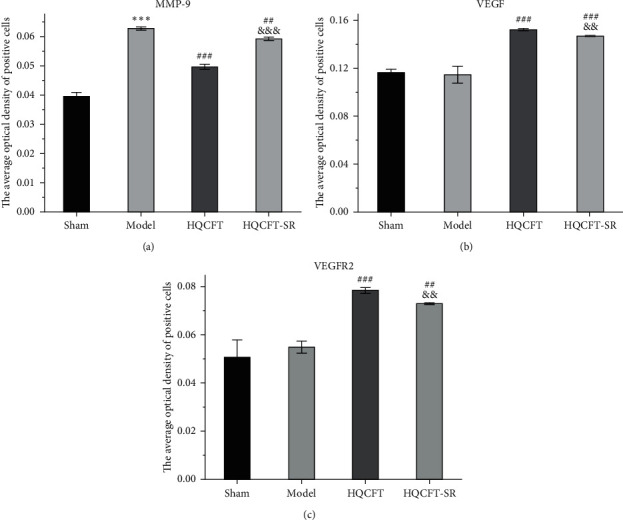
The levels of MMP-9, VEGF, and VEGFR2 in the rat brain tissue. (a) The optical densities of MMP-9-positive cells. (b) The optical densities of VEGF-positive cells. (c) The optical densities of VEGFR2-positive cells. Images were processed and analyzed with ImageJ software. Data are expressed as the mean ± SD (*n* = 3). ^∗∗∗^*p* < 0.001 vs. the sham group, ^##^*p* < 0.01 and ^###^*p* < 0.001 vs. the model group, ^&&^*p* < 0.01 and ^&&&^*p* < 0.001 vs. the HQCFT group.

The MMP-9-, VEGF-, and VEGFR2-positive cells in normal brain tissue are brown, dark brown, or gray. As shown in Figures 6 and 7(a), compared with that in the sham group (0.040 ± 0.001), the average optical density of positive cells in the model group (0.063 ± 0.001) was increased significantly. Compared with the model group, the HQCFT (0.050 ± 0.001) and HQCFT-SR (0.059 ± 0.001) treatment groups showed a marked decrease in the average optical density of positive cells in the brain tissue, and there was a significant difference between those two groups (*p* < 0.001).

As shown in Figures 6, 7(b), and 7(c), the average optical density of VEGF- and VEGFR2-positive cells in the sham group was 0.116 ± 0.003 and 0.051 ± 0.007, respectively. The corresponding values in the model group were 0.115 ± 0.007 and 0.055 ± 0.003, respectively, but there were no statistically significant differences compared with the sham group (*p* > 0.05). Compared with that in the model group, the average optical density of VEGF-positive cells was increased by 31.01% (*p* < 0.001) and 26.23% (*p* < 0.001) in the HQCFT and HQCFT-SR groups, respectively, and the average optical density of VEGFR2-positive cells was increased by 54.99% (*p* < 0.001) and 44.42% (*p* < 0.01) in the HQCFT and HQCFT-SR groups, respectively. The average optical densities of positive cells in the HQCFT group were significantly higher than those in the HQCFT-SR group (*p* < 0.01). The above results indicated that the SR in HQCFT could promote angiogenesis in the brain tissue of rats subjected to MCAO.

### 3.7. Effect of SR on the HIF-1*α*, NLRP3, Caspase 1, IL-1*β*, IL-6, and TNF-*α* Contents in the Serum of Rats Subjected to MCAO

**Figure 8:**
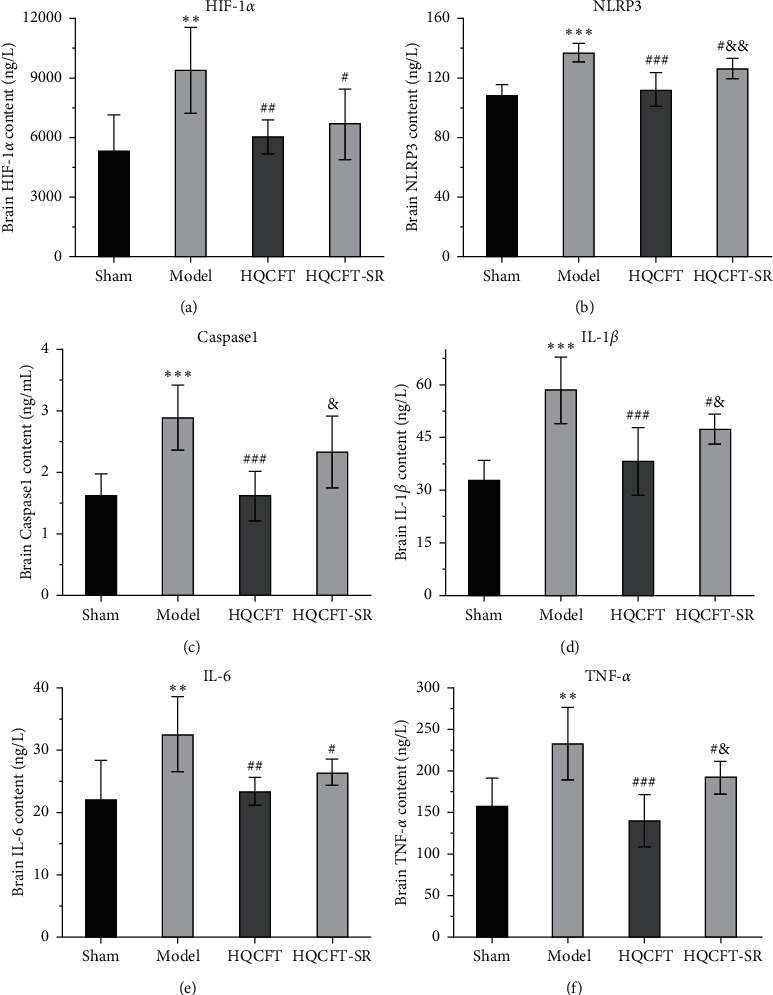
Effects of HQCFT and HQCFT-SR on the activity of HIF-1*α*, NLRP3, caspase 1, IL-1*β*, TNF-*α,* and IL-6 in the serum of rats subjected to MCAO. (a) HIF-1*α* content in rat serum. (b) NLRP 3 content in rat serum. (c) Caspase 1 content in rat serum. (d) IL-1*β* content in rat serum. (e) IL-6 content in rat serum. (f) TNF-*α* content in rat serum. Data are expressed as the mean ± SD (*n* = 12). ^∗∗∗^*p* < 0.001 vs. the sham operation group, ^##^*p* < 0.01 and ^###^*p* < 0.001 vs. the model group, and ^&&^*p* < 0.01 and ^&&&^*p* < 0.01 vs. the HQCFT group.

The inflammatory response is the main feature of the pathological mechanism of CI [16]. To further clarify the anti-inflammatory effect of SR in HQCFT, our study explored the levels of HIF-1*α*, NLRP3, caspase 1, IL-1*β*, IL-6, and TNF-*α* in rat serum with ELISA kits. As shown in Figures 8(a)–8(f), the serum contents of HIF-1*α*, NLRP3, caspase 1, IL-1*β*, IL-6, and TNF-*α* in the sham group were 5336.56 ± 1808.12, 108.35 ± 7.72, 1.62 ± 0.36, 32.61 ± 5.80, 22.03 ± 6.35, and 156.83 ± 34.64, respectively. There were significant differences between the sham group and the model group. Compared with those in the sham group, the HIF-1*α*, NLRP3, caspase 1, IL-1*β*, IL-6, and TNF-*α* contents were 6037.98 ± 852.61, 112.61 ± 11.37, 1.62 ± 0.40, 38.12 ± 9.67, 23.38 ± 2.21, and 140.09 ± 31.42, respectively, in the HQCFT group and 6685.32 ± 1781.75, 126.61 ± 6.88, 2.33 ± 0.58, 47.42 ± 4.11, 26.44 ± 2.08, and 192.13 ± 19.56, respectively, in the HQCFT-SR group. The level of these inflammatory factors in the HQCFT group differed significantly from with those in the HQCFT-SR group. These findings indicated that the SR in HQCFT could inhibit the inflammatory response in the serum of rats subjected to MCAO.

## 4. Discussion

CI is a local ischemic condition characterized by decreased cerebral blood flow and vascular embolism [17]. In the ischemic state, brain tissue is insufficiently supplied with oxygen and nutrients due to restrained and reduced blood flow, which results in a series of inflammatory responses, severe oxidative stress, neuronal damage, and apoptosis [18, 19]. CI has a high rate of disability and mortality, the incidence of CI is on the rise, and there is no optimal treatment strategy [11, 20]. In recent years, it has been reported that Chinese herbal medicines and their components, including AR, PR, and SR, can relieve neurological deficits and promote angiogenesis with low toxicity and few side effects, exerting a good neuroprotective effect in the context of CI [21–23].

Astragaloside IV (AS-IV) is the main active component of AR. Many studies have proven that AS-IV can promote neurogenesis and angiogenesis and improve neurological function [24, 25]. In addition, AS-IV negatively modulates endoplasmic reticulum stress-mediated apoptosis in endothelial cells to inhibit the increase in BBB permeability induced by ischemia/reperfusion and decrease the brain infarction ratio [26]. Cycloastragenol is the main metabolite of AS-IV in vivo, and some researchers proposed that cycloastragenol is a bioactive form of AS-IV [27]. According to Li et al., cycloastragenol could inhibit TNF-*α*, IL-1*β*, MMP-9, and NF-*κ*B p65 activity and expression, protect against tight junction and BBB degradation, and prevent nerve cell apoptosis in an MCAO mouse model [28]. Paeoniflorin is a bioactive ingredient from PR that can significantly reduce the protein expression of NLRP3, ASC, caspase 1, procaspase 1, and IL-1*β* and increase the expression of VEGF/VEGF-R in an MCAO rat model [29, 30]. However, the main active components of SR in CI therapy, including cimifugin, prim-O-glucosylcimifugin, and 5-O-methylvisammioside, have rarely been reported. We speculate that AS-IV, cycloastragenol, and paeoniflorin are the potential active components of HQCFT. Nevertheless, a previous study showed that SR could guide or promote the absorption and distribution of other drugs to improve overall drug efficacy [7]. Therefore, the present study explored the anti-inflammatory and angiogenic effects of SR and investigated the underlying mechanism by which SR guides and enhances the efficacy of HQCFT against CI.

MCAO is a typical model for studying CI [9]. In this study, a MCAO model was used to investigate the guiding and enhancing effects of SR on HQCFT in the treatment of CI. The results of TTC staining showed that the HQCFT and HQCFT-SR treatments effectively reduced the brain infarction area and promoted recovery after local infarction. In addition, the protective effect of HQCFT was significantly better than that of HQCFT-SR.

Lesions of neuronal cells, a feature of ischemic diseases, are usually used to evaluate the extent of CI [31]. Our results confirmed that HQCFT improved the pathological state in rats subjected to MCAO, and cerebral tissue slice experiments verified this finding. In the model group, neurons exhibited disorder and local necrosis; the cells showed severe edema, and the nuclei were pyknotic. However, the neurons in the HQCFT and HQCFT-SR groups were markedly improved, cell edema was relieved, the cells were arranged close to each other, and the nuclei were round.

HIF-1*α* is involved in the pathological process and inflammatory reactions associated with ischemic disease [32]. In the present study, compared with that in the sham group, the content of HIF-1*α* in the HQCFT and HQCFT-SR groups was reduced. As an important inflammatory regulator of CI, HIF-1*α* can stimulate the activation of the NLRP3 inflammasome [12]. However, overactivation of the NLRP3 inflammasome triggers a series of caspase 1 signaling cascades and intensifies the release of IL-1*β* and IL-18, leading to the formation of a vicious cycle and aggravating CI [33–35]. Additionally, IL-6 and TNF-*α* play a crucial role in the inflammatory responses induced by CI [13].

HQCFT and HQCFT-SR attenuate the abnormal expression of NLRP3, caspase 1, IL-1*β*, and IL-6 in brain tissues from rats subjected to MCAO. However, TNF-*α* protein expression levels in the treatment groups were not significantly different from those in the model group. Moreover, HQCFT and HQCFT-SR reduced serum HIF-1*α*, NLRP3, caspase 1, IL-1*β*, IL-6, and TNF-*α* levels. Simultaneously, the contents of these inflammatory factors in the HQCFT-SR group obviously differed from those in the HQCFT group. In summary, these findings suggested that SR can improve the overall anti-inflammatory effect of HQCFT and confirmed that HQCFT-mediated inhibition of inflammation after CI exerts beneficial effects in the cerebrum.

MMP-9 is a matrix metalloproteinase that participates in neuroinflammatory responses and angiogenesis [36]. In the absence of negative regulation, the continuous increase in MMP-9 expression and activity will stimulate the production of inflammatory cytokines, inducing brain edema, blocking angiogenesis, and aggravating ischemic injury [37, 38]. To date, many researchers have regarded MMP-9 as an important target for the treatment of stroke, CI, cardiomyopathy, and other ischemic diseases [39–41]. In this study, we determined the expression level of MMP-9 in the rat brain by Western blotting and immunohistochemistry. The results showed that MMP-9 protein expression levels were significantly reduced in the HQCFT and HQCFT-SR treatment groups. These results indicated that MMP-9 may be an effective target via which SR enhances the efficacy of HQCFT in the treatment of CI.

VEGF is the most important regulatory factor involved in vascular system development and differentiation [42]. VEGF can stimulate angiogenesis and modulate vascular permeability, playing a direct neuroprotective role and promoting neurogenesis to mediate recovery in the context of cerebral ischemic disease [43]. A previous study showed that VEGF improved the effect of cerebral ischemia on the brain parenchyma by activating VEGFR2 in brain endothelial cells and neural stem cells [44]. However, overexpression of MMP-9 could inhibit the production of VEGF and further prevent neurogenesis and angiogenesis [38]. In this study, HQCFT and HQCFT-SR increased the expression of both VEGF and VEGFR2. Interestingly, the expression levels of VEGF and VEGFR2 in the HQCFT treatment group were significantly higher than those in the HQCFT-SR treatment group. The significant upregulation of those indicators in the HQCFT group further proved that SR plays a role in guiding and enhancing the efficacy of HQCFT.

The present study has several limitations. Although we verified that SR could guide or enhance the overall effect of HQCFT on CI in a rat model, whether it has the same effect in other animal models needs further study. In addition, traditional Chinese herbal formulas typically have multiple components, multiple pathways, and multiple targets, but which component of SR exerts a guiding effect on HQCFT needs to be clarified.

In summary, HQCFT and HQCFT-SR are potentially useful in the treatment of CI because they promote angiogenesis and exert anti-inflammatory activity. Importantly, the cytokines in brain tissue and serum from the HQCFT group exhibited better efficacy than those from the HQCFT-SR group. Therefore, SR can improve the effect of HQCFT on CI, and the mechanism by which SR guides and enhances the effect of HQCFT warrants further study.

## Data Availability

The data that support the results of this study are available from the corresponding author upon request.

## Supplementary Materials

Supplementary Materials Graphical abstract: SR enhances the efficacy of HQCFT in the treatment of cerebral infarction by exerting anti-inflammatory effects and promoting angiogenesis.
