# Supplementary material for: Saposhnikoviae Radix Enhanced the Angiogenic and Anti-Inflammatory Effects of Huangqi Chifeng Tang in a Rat Model of Cerebral Infarction
Source: Evid Based Complement Alternat Med. 2021 Sep 21;2021:4232708. doi: 10.1155/2021/4232708 (PMC8478555; doi:10.1155/2021/4232708)
Supplement: Supplementary Materials — Graphical abstract: SR enhances the efficacy of HQCFT in the treatment of cerebral infarction by exerting anti-inflammatory effects and promoting angiogenesis. [file 4232708.f1.docx]

Graphical Abstract


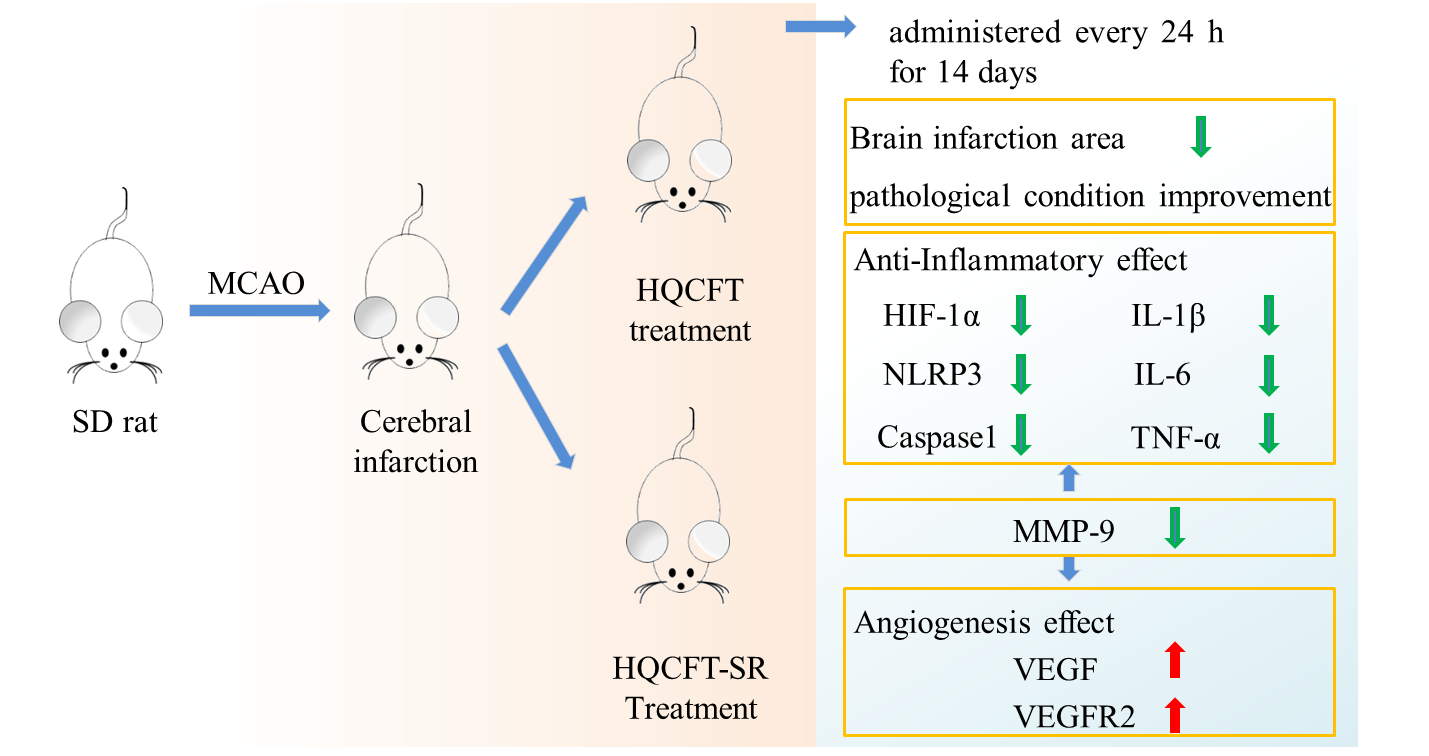


SR enhances the efficacy of HQCFT in the treatment of cerebral infarction via anti-inflammatory and promoting angiogenesis
